# Mitigating the risk of Zika virus contamination of raw materials and cell lines in the manufacture of biologicals

**DOI:** 10.1099/jgv.0.000995

**Published:** 2017-12-14

**Authors:** Joanna Zmurko, Douglas B. Vasey, Claire L. Donald, Alison A. Armstrong, Marian L. McKee, Alain Kohl, Reginald F. Clayton

**Affiliations:** ^1^​ Merck KGaA, BioReliance® Services, Todd Campus, West of Scotland Science Park, Glasgow G20 OXA, UK; ^2^​ MRC-University of Glasgow Centre for Virus Research, Glasgow G61 1QH, UK

**Keywords:** infectivity, cytopathic effect, Zika virus, raw materials, virus detection, virological safety of biologicals

## Abstract

Ensuring the virological safety of biologicals is challenging due to the risk of viral contamination of raw materials and cell banks, and exposure during in-process handling to known and/or emerging viral pathogens. Viruses may contaminate raw materials and biologicals intended for human or veterinary use and remain undetected until appropriate testing measures are employed. The outbreak and expansive spread of the mosquito-borne flavivirus Zika virus (ZIKV) poses challenges to screening human- and animal -derived products used in the manufacture of biologicals. Here, we report the results of an *in vitro* study where detector cell lines were challenged with African and Asian lineages of ZIKV. We demonstrate that this pathogen is robustly detectable by *in vitro* assay, thereby providing assurance of detection of ZIKV, and in turn underpinning the robustness of *in vitro* virology assays in safety testing of biologicals.

## Introduction

The risk of contamination cell lines and raw materials from emerging viruses is of great importance in the production of biopharmaceuticals. Globalization is not limited to the flow of money, services, goods and people across geographic boundaries, but also includes the distribution of pathogens into non-indigenous locations [1]. As a result, in recent decades the emergence and re-emergence of viruses from one geographical location to another, or from one susceptible host to another, has been observed [2–4]. Human immunodeficiency virus, severe acute respiratory syndrome coronavirus, Middle East respiratory syndrome coronavirus, influenza virus (type A, H1N1), hepatitis A virus, Ebola virus, West Nile virus (WNV) and Zika virus (ZIKV) are examples of virus species whose incidence and geographical spread has increased in the past 20 years and could further diversify in the near future. The development and manufacture of biologicals and advanced therapy medicinal products (ATMPs) employs various cell lines, raw materials and active pharmaceutical ingredients (APIs) [5] from human and animal sources that require sophisticated safety assays and Good Manufacturing Practice (GMP) standards. Potential viral contaminants, including emerging viruses, may enter biological production processes from cell lines or raw materials thus posing a risk to downstream applications and to drug safety. The risk of viral contamination must therefore be mitigated by stringent testing at the appropriate stages of production. Regulatory guidelines and recommendations on viral safety testing outline requirements for testing at various stages of a product life cycle, including raw materials, cell substrates, virus seeds, unprocessed bulk harvests and end products.

**Fig. 1.**
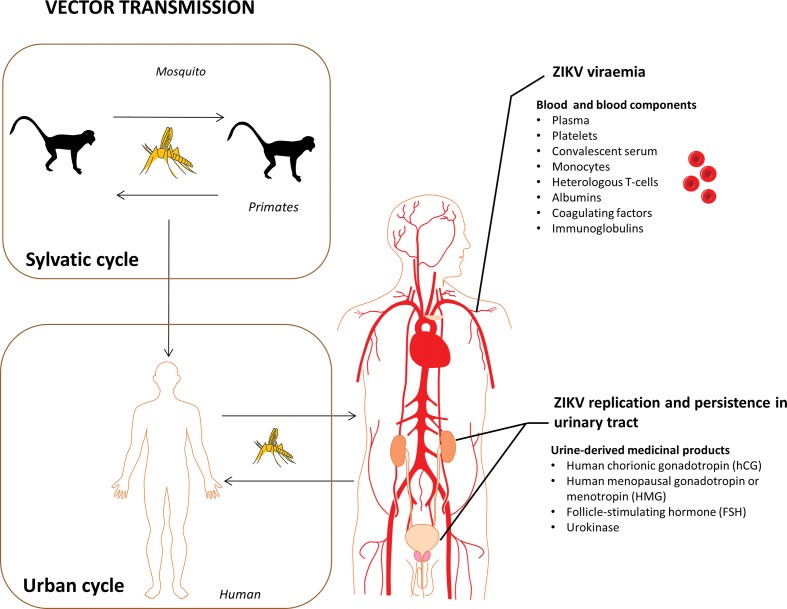
Transmission of Zika virus (ZIKV) and risk for manufacturing of biologicals and advanced therapy medicinal products. In nature, ZIKV circulates through a sylvatic cycle involving multiple mosquito species (primarily from the *Aedes* family) and primates, the reservoir species for ZIKV. The predominant transmission route of ZIKV to humans occurs via the bite of an infected mosquito. Infectious ZIKV particles have been detected in the blood, serum and urine of infected humans. Infection with ZIKV in the majority (~80–90 %) of healthy humans is asymptomatic. As a result, ZIKV may enter biopharmaceutical production in materials from viraemic, yet asymptomatic, donors or from donors with persistent replication of the virus in the urinary tract or renal system. Therefore raw material of human origin from affected areas should be considered as potentially contaminated with ZIKV including, but not limited to, materials such as blood fractions (plasma, platelets, and convalescent serum) and urine, which serves as a source of pharmacologically active substances (human chorionic gonadotropin (hCG), human menopausal gonadotropin or menotropin (HMG), follicle-stimulating hormone (FSH) and urokinase).

ZIKV is an emerging arthropod-borne member of the *Flaviviridae* virus family and is closely related to other mosquito-borne arboviruses such as dengue virus, yellow fever virus, WNV and Japanese encephalitis virus [6–9]. The introduction of ZIKV in the Americas in 2015 [10, 11] highlighted its epidemic potential and global public health burden [12, 13]. Infection with ZIKV in the majority (~80–90 %) of healthy humans is asymptomatic but it can progress to neurological complications, such as encephalitis, meningo-encephalitis, Guillain–Barré syndrome in adults and birth defects, such as fetal and neonatal microcephaly grouped together as congenital Zika syndrome [14–17]. As of 2016 ZIKV is present in many countries in Africa, Asia and across the Americas, causing truly global concern. Despite the identification of ZIKV in 1947 and its reported presence in Africa and Asia for many decades [18], little attention has been paid to its potential to contaminate raw products of human or animal origin used in the manufacture of biologicals. In nature, ZIKV circulates through a sylvatic cycle involving multiple mosquito species (primarily from the *Aedes* family) and primates, the natural reservoir species for ZIKV [19–23] (Fig. 1). The predominant transmission route of ZIKV to humans occurs via the bite of an infected mosquito, although non-vector-borne routes of ZIKV transmission have been documented, including sexual transmission [24–26], blood transfusion [27, 28], organ transplantation [29] and perinatal transplacental transmission [30]. Infectious ZIKV particles have been detected in saliva, blood, serum, semen and urine [25, 28, 31–33] of infected humans. Indeed, the broad tropism of the virus and its persistence [17] may lead to longer-term issues in affected countries. As a result, ZIKV may enter biopharmaceutical production in materials from viraemic, yet asymptomatic, donors or from donors with persistent replication of the virus in the urinary tract or renal system, as the possibility of persistent ZIKV infection in multiple tissues has recently been demonstrated in a rhesus macaque model [34]. Therefore, raw material of human origin from affected areas should be considered as potentially contaminated with ZIKV including, but not limited to, blood and blood components (plasma, platelets, convalescent serum, monocytes, heterologous T cells, albumins, coagulating factors, immunoglobulins) [35] and urine, which serves as a source of pharmacologically active substances such as human chorionic gonadotropin (hCG), human menopausal gonadotropin or menotropin (HMG), follicle-stimulating hormone (FSH) and urokinase [36–38]. Limited epidemiologic surveillance evidence suggests that ZIKV infection in wider mammal species [39] may be possible. A study performed in Indonesia in the late 1970s suggested that horses, cows, carabaos (water buffaloes), goats, ducks and bats were seropositive for antibodies against ZIKV [21, 40]. This may suggest that raw materials from these species may harbour this pathogen, but the limited nature of the study and lack of independent correlation relegates the risk to theoretical. However, it is worth mentioning that even in monkeys and apes, which are natural reservoirs of ZIKV, only a few naturally and experimentally infected monkeys and apes have demonstrated any symptoms or manifested any clinical disease when infected with ZIKV [21]. Furthermore, recent reports of ZIKV replication in cell lines such as non-human primate (Vero and LLC-MK2), pig (PK-15), rabbit (RK-13), hamster (BHK21) and chicken (DF-1) [41] clearly suggest that a wide range of animal cell lines relevant to the manufacture of biopharmaceuticals may be susceptible to ZIKV. In line with a report that goats were seropositive for antibodies against ZIKV, there is a theoretical risk of the introduction of ZIKV contamination during pharmaceutical processes using raw materials of animal origin. This includes clonal selection of cells lines by cell sorting with antibodies or antisera from animal species (goats). To mitigate the risk of viral contamination, biological materials are tested by *in vitro* assays for the detection of adventitious viruses, using several susceptible detector cell lines where the classical reports of cytopathic effects (CPE), haemadsorption and haemagglutination [42, 43] may be observed. We have recently demonstrated that Schmallenberg orthobunyavirus, an emerging viral pathogen of cattle and sheep, is detectable by classical *in vitro* adventitious virus assays [44]. ZIKV has been reported to elicit CPE in a panel of continuous cell lines [45, 46], leading us to suggest that an *in vitro* assay utilizing appropriate cells lines with CPE end point, in a GMP-compliant assay platform, may represent a suitable means to ensure the robust detection of ZIKV in biological materials. The Food and Drug Administration (FDA) [47], European Pharmacopeia (Ph. Eur) [48] and World Health Organisation (WHO) [49] guidelines for the qualification of cell substrates and other raw materials used for the production of biologicals specify that the monolayer cultures of detector cells include cultures of the same species and tissue type used for production of the test article, in addition to cultures of a human diploid cell line and monolayer cultures of another cell line of a different species (FDA requirements specify that a monkey kidney cell line should be used). Therefore, for the purpose of this study, the human diploid cell line MRC-5 (ATCC, CCL 171) and secondary African green monkey kidney cells, Vero (ATCC, C1008 and ATCC, CCL-81) were challenged with two strains of ZIKV (MR766 and PE243) as detector cells during classical *in vitro* adventitious virus biosafety assay.

## Results

Cell lines used in this study were manufactured and thoroughly characterized under GMP conditions according to guidelines of the International Conference on Harmonization Topic Q5 (ICH Q5) [50]. The characterization of MRC-5 and Vero cells includes confirmation of cell line identity and the assessment of purity and freedom from fungi and bacteria (sterility), as well as mycoplasma and adventitious viruses testing.

**Fig. 2.**
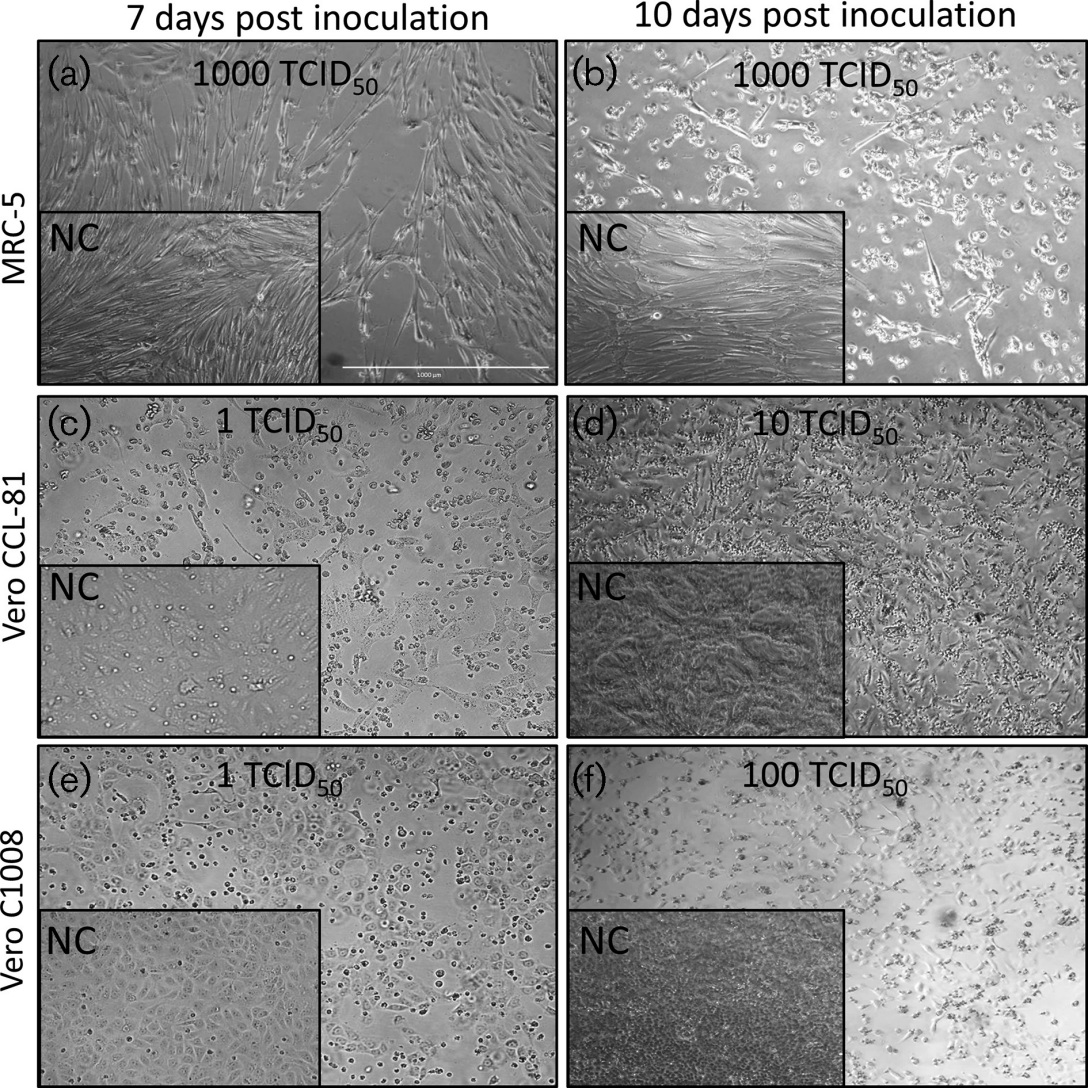
Cytopathic effects observed in detector cell lines challenged with ZIKV MR766. Monolayers of detector cells were incubated with 1000, 100, 10 and 1 TCID_50_ ZIKV MR766 monitored for CPE over a period of 14 or 28 days. Micrographs shown are representative of the observations made in detector cell lines following ZIKV MR766 inoculation and days p.i. (dpi) (panels a, b, c, d, e and f). CPE in the form of retarded cell growth and extensive cell rounding and lysis was observed. Text in each panel indicates the detector cell line identity and dpi on which the image was recorded. Inset panels show negative control (NC) mock-infected cells. Scale bar is 1000 µm.

**Fig. 3.**
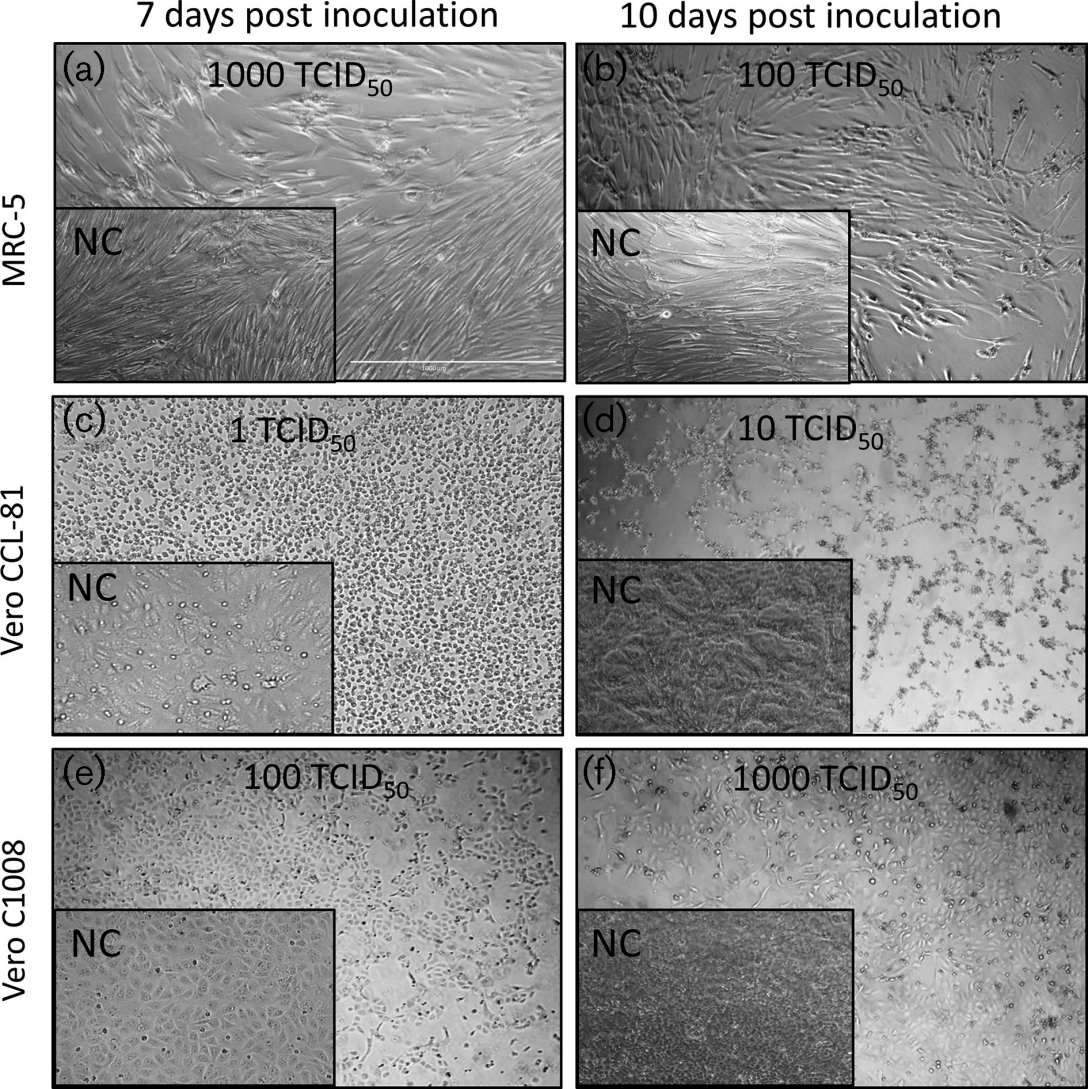
Cytopathic effects observed in detector cell lines challenged with ZIKV PE243. Monolayers of detector cells were incubated with 1000, 100, 10 and 1 TCID_50_ ZIKV PE243 and monitored for CPE over a period of 14 or 28 days. Micrographs shown are representative of the observations made in detector cell lines following ZIKV PE243 inoculation and days p.i. (dpi) (panels a, b, c, d, e and f). CPE in the form of retarded cell growth and extensive cell rounding and lysis was observed. Text in each panel indicates the detector cell line identity and dpi on which the image was recorded. Inset panels show negative control (NC) mock-infected cells. Scale bar is 1000 µm.

The human diploid cell line MRC-5 demonstrated cytopathic effects (CPE) when inoculated with 1000, 100, 10 and 1 TCID_50_ of ZIKV MR766, an African lineage virus (Fig. 1a, b and Table 1). The structural changes to host cell morphology, CPE in the form of retarded cell growth and extensive cell rounding and lysis by ZIKV were clearly observed. Inoculation of MRC-5 with 1000 TCID_50_ of ZIKV MR766 was reported in the majority of wells between days 6 and 10 post inoculation (pi) with the mean appearance of CPE on day 8. MRC-5 cells demonstrated early signs of CPE following inoculation with 100 TCID_50_ ZIKV MR766 at 6 days post infection (p.i.), and CPE was observed in all wells at day 14 p.i. with the mean appearance of CPE at day 10. When inoculated with 10 TCID_50_ of ZIKV MR766, CPE was reported between days 14 and 17 p.i. with the mean appearance at day 13. Inoculation with 1 TCID_50_ ZIKV MR766 reported CPE in 33 % of wells on day 14 and in 50 % of wells on day 28 (Table 4). The inoculation of the Brazilian isolate (Asian lineage) ZIKV PE243[51] at levels of 1000, 100, 10 and 1 TCID_50_ on the MRC-5 detector cell line elicited CPE in 67, 44–27 and 11 % of wells, respectively, during a 14-day *in vitro* assay (Tables 2 and 4, Fig. 3a, b). The mean appearance of CPE for 1000 and 100 TCID_50_ was at days 12 and 14 p.i. On day 14, supernatant from MRC-5 was inoculated onto a subconfluent monolayer of MRC-5 and CPE was observed in 67, 75, 42 and 17 % of wells, respectively, at day 28. CPE in Vero C1008 and Vero CCL-81 cells was similar to that observed on MRC-5 cells and presented as structural changes to host cell morphology, retarded cell growth and extensive cell rounding, detachment and lysis. Vero C1008 reported the presence of 1000 and 100 TCID_50_ of ZIKV MR766 on all occasions at 14 days *in vitro* assay. The onset of CPE was observed as early as 3 days p.i. with the mean appearance of CPE on days 5 and 7 for 1000 and 100 TCID_50_, respectively. ZIKV MR766 at 10 and 1 TCID_50_ elicited CPE in 67 and 56 % of Vero C1008 wells during 14 days of *in vitro* culture, with the mean appearance of CPE on days 8 and 9. Vero C1008 detected the presence of 1000,100, 10 and 1 TCID_50_ of ZIKV MR766 in each well inoculated on all occasions during the 28-day *in vitro* assay (Tables 2 and 4, Fig. 2e, f). Inoculation with 1000 and 100 TCID_50_ of ZIKV PE243 elicited CPE in Vero C1008 wells on all occasions during the 14 days of *in vitro* assay, with the mean appearance of CPE on day 8. Vero C1008 detected the presence of 10 and 1 TCID_50_ of ZIKV PE243 on 67 and 56 % occasions during the 14 days of *in vitro* assay, with the mean appearance of CPE for 10 TCID_50._ reported on day 9. Inoculation with 1000, 100 and 10 TCID_50_ ZIKV PE243 elicited CPE in Vero C1008 wells on all occasions during the 28 days of *in vitro* assay. Inoculation with 1 TCID_50_ of ZIKV PE243 reported CPE in 83 % of wells on day 28 (Tables 2 and 4, Fig. 3e, f). Vero CCL-81 detected 1000 and 100 TCID_50_ of ZIKV MR766 in all wells inoculated on each occasion, in 94 % of wells inoculated with 10 TCID_50_ ZIKV MR766 and in 44 % of wells inoculated with 10 TCID_50_ in a 14-day *in vitro* assay (Tables 3 and 4). The mean appearance of CPE was reported on day 6 for 1000 TCID_50_ inoculum, on day 7 for 100 TCID_50_ and on day 8 for 10 and 1 TCID_50_. Vero CCL-81 detected 1000, 100 and 10 TCID_50_ of ZIKV MR766 in all wells inoculated on all occasions and in 75 % of wells inoculated with 1 TCID_50_ during a 28-day *in vitro* assay (Tables 3 and 4, Fig. 2c, d). Inoculation with 1000, 100, 10 TCID_50_ of ZIKV PE243 was detected on all occasions in a 14-day *in vitro* assay. ZIKV PE243 inoculated at 1 TCID_50_resulted in CPE in 39 % of inoculated Vero CCL-81 detector cells in a 14-day *in vitro* assay and in 100 % of inoculated cells in a 28-day *in vitro* assay (Tables 3 and 4, Fig. 3c, d). The mean appearance of CPE was reported on day 6 for 1000 TCID_50_ inoculum, on day 7 for 100 TCID_50_, on day 9 for 10 TCID_50_ and on day 14 for 1 TCID_50_. Inoculation of detector cells with measles virus and bovine parainfluenza virus type 3 at 100 TCID_50_, representing system suitability controls for MRC-5 and Vero detector cell lines, respectively, elicited clear detection on all occasions (*n*=18) within 6 days (Table 4).

**Table 1.** Microscopic detection of CPE observed on MRC-5 cells challenged with strains of ZIKV Monolayers of detector cells were incubated with 1000, 100, 10 and 1 TCID_50_ ZIKV MR766 and ZIKV PE243 and monitored for CPE over a period of 28 days. Data represent viral inoculations performed on 3 separate occasions (runs 1–3) for each dilution (1000, 100, 10 and 1 TCID_50_) of ZIKV MR766 and PE243 on MRC-5 cells. On day 14, supernatants from cultures not showing CPE were inoculated onto fresh detector cells (runs 1 and 2). Data in the table indicate the first day of CPE appearance, median and mean appearance of CPE, standard variation and coefficient of variation. Statistical outliers were identified and removed from the analysis.

**Day of CPE appearance on MRC-5 cells**									
**Run**	**Well**	**ZIKV MR766**				**ZIKV PE243**			
		**1000 TCID_50_**	**100 TCID_50_**	**10 TCID_50_**	**1 TCID_50_**	**1000 TCID_50_**	**100 TCID_50_**	**10 TCID_50_**	**1 TCID_50_**
1	1	7	7	10	–	7	10	10	10
	2	7	7	14	–	7	10	10	10
	3	7	7	17	–	10	10	10	–
	4	7	7	17	–	10	10	10	–
	5	7	14	17	–	10	20	10	–
	6	10	14	17	–	10	20	–	–
2	7	10	10	10	10	–	20	–	–
	8	10	10	10	10	–	20	–	–
	9	10	10	10	10	–	–	–	–
	10	10	14	10	14	–	–	–	–
	11	10	14	14	14	–	–	–	–
	12	10	14	14	14	–	–	–	–
3	13	3	7	10	n/a	14	10	n/a	n/a
	14	7	7	14	n/a	14	10	n/a	n/a
	15	7	7	14	n/a	14	10	n/a	n/a
	16	10	10	n/a	n/a	14	14*	n/a	n/a
	17	10	10	n/a	n/a	14	n/a	n/a	n/a
	18	10	10	n/a	n/a	14	n/a	n/a	n/a
Median		9	9	12	nd	12	13	nd	nd
Mean		8	10	13	nd	12	14	nd	nd
Standard variation		1	1	2	nd	3	4	nd	nd
Coefficient of variation (%)		13	15	13	nd	22	29	nd	nd

–, No CPE was visible; n/a, non-applicable; nd, not determined.

*Statistical outlier.

**Table 2.** Microscopic detection of cytopathic effect observed on Vero C1008 cell line challenged with ZIKV Monolayers of detector cells were incubated with 1000, 100, 10 and 1 TCID_50_ ZIKV MR766 and ZIKV PE243 and monitored for CPE over a period of 28 days. Data represent viral inoculations performed on 3 separate occasions (runs 1–3) for each dilution (1000, 100, 10 and 1 TCID_50_) of ZIKV MR766 and PE243 on Vero C1008 cells. On day 14, supernatants from cultures not showing CPE were inoculated onto fresh detector cells (runs 1 and 2). Data in the table indicate the first day of CPE appearance, median and mean appearance of CPE, standard deviation and coefficient of variation. Statistical outliers were identified and removed from the analysis.

**First day of CPE appearance on Vero C1008**									
**Run**	**Well**	**ZIKV MR766**				**ZIKV PE243**			
		**1000 TCID_50_**	**100 TCID_50_**	**10 TCID_50_**	**1 TCID_50_**	**1000 TCID_50_**	**100 TCID_50_**	**10 TCID_50_**	**1 TCID_50_**
1	1	3	7	7	7	7	7	7	7
	2	3	7	7	7	7	7	7	7
	3	3	7	7	7	7	7	7	7
	4	3	7	7	7	7	7	7	7
	5	3	7	7	7	7	7	7	–
	6	3	7	7	7	7	7	24*	–
2	7	3	3	3	7	7	3	7	3*
	8	3	3	3	7	7	3	7	7
	9	3	3	7	7	7	3	7	7
	10	7	7	7	7	7	3	7	7
	11	7	7	7	20	7	3	7	7
	12	7	7	20*	20	7	7*	7	7
3	13	3*	3*	10	–	7	14	14	–
	14	7	10	10	–	7	14	14	–
	15	7	10	14	–	7	14	14	–
	16	7	10	14	–	10	14	14	–
	17	7	10	14	–	10	14	14	–
	18	7	10	14	–	10	14	14	–
Median		5	7	8	7	8	8	9	7
Mean		5	7	8	9	8	8	9	7
Standard deviation		2	2	3	2	1	5	3	0
Coefficient of variation (%)		33	28	37	24	9	57	35	0

–, No CPE was visible; n/a, non-applicable.

*Statistical outlier.

**Table 3.** Microscopic detection of cytopathic effect observed on Vero CCL-81 cell line challenged with ZIKV Monolayers of detector cells were incubated with 1000, 100, 10 and 1 TCID_50_ ZIKV MR766 and ZIKV PE243 and monitored for CPE over a period of 28 days. Data represent viral inoculations performed on 3 separate occasions (runs 1–3) for each dilution (1000, 100, 10 and 1 TCID_50_) of ZIKV MR766 and PE243 on Vero CCL-81 cells. On day 14, supernatants from cultures not showing CPE were inoculated onto fresh detector cells (runs 1 and 2). Data in the table indicate the first day of CPE appearance, median and mean appearance of CPE, standard deviation and coefficient of variation. Statistical outliers were identified and removed from the analysis.

**First day of CPE appearance on Vero CCL-81**									
**Run**	**Well**	**ZIKV MR766**				**ZIKV PE243**			
		**1000 TCID_50_**	**100 TCID_50_**	**10 TCID_50_**	**1 TCID_50_**	**1000 TCID_50_**	**100 TCID_50_**	**10 TCID_50_**	**1 TCID_50_**
**1**	1	3	7	7	7	3	7	7	7
	2	3	7	7	7	3	7	7	7
	3	3	7	7	7	3	7	7	7
	4	3	7	7	7	3	7	7	7
	5	3	7	14	14	3	7	7	7
	6	3	7	17	17	3	7	7	7
**2**	7	7	7	7	7	3*	7	7	7
	8	7	7	7	7	7	7	7	20
	9	7	7	7	7	7	7	7	20
	10	7	7	7	–	7	7	7	20
	11	7	7	7	–	7	7	14	20
	12	7	7	7	–	7	7	14	20
**3**	13	7	7	7	n/a	7	7	7	n/a
	14	7	7	7	n/a	7	7	7	n/a
	15	7	7	7	n/a	7	7	10	n/a
	16	7	7	10	n/a	7	7	10	n/a
	17	7	7	10	n/a	7	7	14	n/a
	18	7	7	10	n/a	7	7	14	n/a
Median		6	7	7	7	6	7	8	14
Mean		6	7	8	8	6	7	9	14
Standard deviation		2	0	1	1	2	0	1	7
Coefficient of variation (%)		33	0	14	17	33	0	16	48

–, No CPE was visible; n/a, non-applicable.

*Statistical outlier.

**Table 4.** Summary table indicating percentage of detector cells reporting CPE at days 14 and 28 post infection Microscopic detection of CPE observed on MRC-5 cells, Vero C1008 and Vero CCL-81 challenged with strains of ZIKV. Monolayers of detector cells were incubated with 1000, 100, 10 and 1 TCID_50_ ZIKV MR766 and ZIKV PE243 and monitored for CPE over a period of 28 days. On day 14, supernatants from cultures with less than 100 % of the wells showing CPE were inoculated onto fresh detector cells. Inoculation of detector cells with measles virus and bovine parainfluenza virus type 3 at 100 TCID_50_ represents system suitability controls for MRC-5 and Vero detector cell lines, respectively.

**Inoculum**	**Virus inoculation**		**MRC-5**		**Vero C1008**		**Vero CCL-81**	
	**TCID_50_ per well**	**Infectious units** (λ) present*	**Day 14**	**Day 28**	**Day 14**	**Day 28**	**Day 14**	**Day 28**
Negative control	n/a	n/a	0 %	0 %	0 %	0 %	0 %	0 %
ZIKV MR766	1000	690	100 %	100 %	100 %	100 %	100 %	100 %
	100	69	100 %	100 %	100 %	100 %	100 %	100 %
	10	6.9	61 %	100 %	67 %	100 %	94 %	100 %
	1	0.69	33 %	50 %	33 %	100 %	44 %	75 %
ZIKV PE243	1000	690	67 %	67 %	100 %	100 %	100 %	100 %
	100	69	44 %	75 %	100 %	100 %	100 %	100 %
	10	6.9	27 %	42 %	67 %	100 %	100 %	100 %
	1	0.69	11 %	17 %	56 %	83 %	39 %	100 %
Measles virus	100	69	100 %	100 %	n/a	n/a	n/a	n/a
PI3	100	69	n/a	n/a	100 %	100 %	100 %	100 %

n/a, non-applicable.

*A TCID_50_ unit is the amount of virus required to result in infection of one-half of the cultures inoculated. Thus, for a virus stock at a concentration of 1 TCID_50_ unit/ml, the probability that a well inoculated with 1 ml will be uninfected is 0.5 (and is the same as the probability that it will be infected; i.e. *P*=0.5 for both). Therefore, where a volume contains 1 TCID_50_ unit, the number of infection units present is λ=-ln (0.5) OR ln (2). Thus, 1 TCID_50_ unit is equivalent to ln (2) infectious units (or ~0.69 infectious units).

## Discussion

In this study we addressed the use of a classical *in vitro* assay with CPE end-point testing for the detection of ZIKV contamination in biologicals. The Ph. Eur, sections 2.6.16 [42] and 5.2.3 [48] recommend that cell culture testing for adventitious viral contaminants on viral vaccines for human use is performed for a minimum of 14 days. Furthermore it is also recommended that veterinary vaccines are tested for the presence of adventitious viruses in an *in vitro* assay with a minimum of 28 days' culture (Ph. Eur. Section 5.2.4) [43]. In this study we challenged detector cell lines with ZIKV of African (MR766) and Asian lineages (PE243) during 14- and 28-day *in vitro* assays to assess standard GMP guidelines for adventitious virus testing. We report that the general *in vitro* adventitious virus test with a CPE end point, as used in GMP-compliant safety testing of biologicals, was demonstrably suitable for robust and reproducible detection (100 % detection during our study) of single-digit levels of ZIKV. Many flaviviruses cause haemadsorption and haemagglutination with certain types of erythrocytes [52]. Although not addressed in our study, haemadsorption and haemagglutination end-point tests could serve as suitable and complementary to the CPE method of detecting ZIKV following 14- and 28-day *in vitro* culture. However, where haemagglutination end-point tests are known to be concentration dependent *in vitro* [53], clear and unambiguous CPE offers a robust end point that does not rely on species-dependent erythrocyte selection.

Diverse phenotypes of ZIKV have been demonstrated [41, 54, 55] where African strains showed a higher infection rate than Asian strains, and varying yields of virus produced in human neuronal cell lines were recently reported. In the context of quality assurance and GMP compliance it is therefore also important to assess whether strains from different origins are robustly detectable, and the variation in susceptibility of detector cell lines must be taken into account when assessing the presence of ZIKV in biologicals. We therefore assessed the suitability of two cell lines, the human diploid MRC-5 and African green monkey Vero cell line, to detect ZIKV of African and Asian lineage. MRC-5 cells robustly and reproducibly detected single-digit levels of ZIKV MR766 whereas inoculation with ZIKV PE243 was detected on some, but not all, occasions. In order to assess the suitability of Vero cells to detect different lineages of ZIKV, we tested two subtypes of Vero cells that are currently in use in GMP testing laboratories. First, Vero (ATCC CCL-81), which were isolated from an African green monkey (*Chlorocebus* sp.) in 1962 and are referred to as ‘the original Vero cells’ [56]. Second, Vero C1008 (ATCC CRL-1586), which are a clone of Vero 76 (ATCC CRL-1587) isolated from Vero CCL-81 in 1968. Vero C1008 cells exhibit a slower growth rate in comparison to Vero CCL-81 and show some contact inhibition, and are thus suitable for the propagation and detection of slowly replicating viruses, or viruses which may require several rounds of replication before becoming fit and detectable for replication in cell culture. In our study Vero CCL-81 and Vero C1008 cells both indicated the presence of ZIKV strains MR766 and PE243 in a similar manner. At day 14 of the assay, 10 TCID_50_ of ZIKV MR766 was detected on 67 % of Vero C1008 and 94 % of Vero CCL-81 wells, and 10 TCID_50_ of ZIKV PE243 on 67 % of Vero C1009 and 100% Vero CCL-81 wells. Detection of isolates from African and Asian lineage down to the level of 10 TCID_50_ (approximately 6.9 infectious virus particles) was observed on all occasions in all inoculated wells for both Vero cell lines at day 28. Inoculation with only 1 TCID_50_ (approximately 0.69 infectious virus particles) was indicated by CPE on 33–44 % of wells on day 14 and on 75–100 % of wells on day 28 of the assay (Table 4). Our data show that that when testing for the presence of ZIKV in biologicals is required, Vero cells detect ZIKV contamination robustly and reproducibly with a suitable level of sensitivity. We acknowledge that other strains of ZIKV (e.g. from central Asia – Cambodia and Thailand) may show varying phenotypes during *in vitro* culture which may also depend on the innate competencies of the cell line in restricting viruses. However for the purposes of this work, two divergent strains representing the two major lineages have been selected. Vero cell lines are deficient in the synthesis of interferon alpha and beta (IFN α/β) – host immune defence molecules aimed at restricting viral replication, and a wide range of arthropod-borne viruses including strains of dengue types 1–4, West Nile, Japanese encephalitis, Usutu [57, 58] and ZIKV [41] replicate and elicit CPE in Vero cells. Importantly, due to an ability to support replication of a wide range of flaviviruses, the Vero CCL-81 line is also recommended for virus stock production in the EU Horizon 2020-funded ZIKAlliance consortium [59] and used by the Public Health England National Collection of Pathogenic Viruses (NCPV) to generate authenticated stocks of ZIKV for supply [60].

The aim of the present study was to determine whether a generically validated assay (*in vitro* adventitious virus assay) is able to detect ZIKV that might be present in the raw materials and cell lines used for the production of biologicals. We demonstrated that this pathogen is robustly detectable by *in vitro* assay, thereby providing assurance of detection of ZIKV and in turn underpinning the robustness of *in vitro* virology assays in safety testing of biologicals. Importantly, the matrix of the product may be inhibitory to virus detection during the assay and consequently affect the sensitivity of the detection. Therefore one of the key assessments during biosafety testing for finished biologicals is to establish the level of effect, if any, of the test article matrix on a generally validated assay to detect contaminants. A study known as a Product Specific Qualification (PSQ) examines and quantitates the effect of representative batches of a defined production process on the performance of an assay. Matrices of bulk harvest that may be ostensibly similar may have quite different behaviour during *in vitro* assay, and PSQs address this. A PSQ is normally performed before or during phase III clinical development and is required as a part of the Biologicals Licence Application.

In summary, we demonstrated the robust detection of ZIKV using classical *in vitro* assays for the detection of adventitious viruses with MRC-5 and Vero cells. We demonstrated robust detection of single digits of ZIKV of African and Asian lineage on Vero cells in a 28-day classical *in vitro* assay with CPE end-point testing. The described study uses a proactive and evidence-based approach for mitigating the risk of ZIKV contamination of raw materials, cell lines and other components used in the manufacture of biologicals.

## Methods

### Cells and viruses

The human diploid cell line, MRC-5 (CCL 171) and secondary African green monkey kidney cells, Vero (C1008 and CCL-81) were obtained from the American Type Culture Collection (ATCC) and manufactured and thoroughly characterized by BioReliance under GMP conditions according to guidelines of the International Conference on Harmonization Topic Q5 (ICH Q5) [50]. The characterization of MRC-5 and Vero cells includes confirmation of cell line identity and the assessment of purity and freedom from fungi and bacteria (sterility), as well as mycoplasma and adventitious viruses testing. MRC-5 cells (lot number: 04112W, passage 29–31) were cultured in high-glucose Dulbeco’s modified Eagle’s medium (HG-DMEM, Gibco) supplemented with 10 % Foetal Clone III (Hyclone), 2 mM l-glutamine (Gibco) and 1 mM non-essential amino acids (NEAA, Gibco). Vero C1008 (lot number: 040711W, passage 30–52) and Vero CCL-81 (lot number: 120606, passage 139–150) cells were cultured in HG-DMEM with 10 % fetal bovine serum (FBS, Gibco). For titration and subsequent *in vitro* adventitious virus assays, both detector cell lines were maintained in Eagle’s minimum essential medium (EMEM, Gibco) supplemented with 2 % FBS, 2 mM l-glutamine, 1 mM sodium pyruvate (Gibco), 100 U ml^−1^ penicillin (Gibco), 100 µg ml^−1^ streptomycin (Gibco), 2.4 µg ml^−1^ amphotericin B (Sigma) and 50 µg ml^−1^ Gentamicin (Gibco). Two strains of ZIKV were used for this study, representing the African and Asian lineages. The African ZIKV strain, LC002520/MR766/1947/Uganda (abbreviated as MR766) was isolated in 1947 from Rhesus monkeys in the Zika Forest in Uganda. ZIKV MR766 was obtained from BEI Resources, NIAID, NIH: Genomic RNA from ZIKV Virus, MR766, NR-50085. The Asian lineage ZIKV strain: ZIKV/*H.sapiens*/Brazil/PE243/2015 (abbreviated to ZIKV PE243), was isolated from a patient from Recife, Brazil in 2015 and produced as described previously [51]; this strain was characterized and is available at the MRC-University of Glasgow Centre for Virus Research. ZIKV MR766 and ZIKV PE243 were titrated on Vero cells (ATCC CCL-81) using 96-well titration with eight replicates for each virus dilution. Each virus was titrated with four replicates performed on two occasions. The mean titre obtained for ZIKV MR766 was 8.88×10^7^ TCID_50_ ml^−1^ (7.89 log_10,_ SD=0.2 log_10,_ *n*=4), and 4.80×10^7^ TCID_50_ ml^−1^ (7.41 log_10,_ SD=0.5 log_10_, *n*=4) for ZIKV PE243. The bovine parainfluenza virus type 3 (PI3, VR-281) and measles virus (VR-24, Edmonston strain) were obtained from ATCC. PI3 and measles viral stocks were produced and titrated according to GMP.

### *In vitro* adventitious virus assay platform

Detector cells (Vero ATCC C-1008, Vero ATCC CCL-81 and MRC-5 ATCC CCL 171) were seeded at 1.0×10^5^ cells ml^−1^ in 2 ml growth medium (10 % HG DMEM) in 6-well plates. Monolayer health and confluency were examined continually post seeding. The medium was removed from all cultures and the cells washed once with approximately 1 ml/well of phosphate buffered saline (PBS, Gibco). For negative controls inoculations, one 6-well plate per cell type was inoculated with maintenance medium (2 % EMEM) using a volume of 0.5 ml per well. Following incubation at 36.5±1.5 °C for 70±10 min, the inoculum was removed and the cultures were refed with 2 ml per well maintenance medium (2 % EMEM). ZIKV was diluted to titres of 2000, 200, 20 and 2 TCID_50_ per ml in 2 % culture medium. One 6-well plate for each cell type was inoculated with each dilution of virus using an inoculum volume of 0.5 ml per well as follows: MRC-5, Vero CCL-81 and Vero C1008 cells were inoculated with 1000, 100, 10 and 1 TCID_50_ of either ZIKV MR766 or ZIKV PE243 separately. Furthermore, MRC-5 cells were inoculated with measles virus at 100 TCID_50_ and Vero cells (CCL-81 and C1008) were inoculated with parainfluenza virus type 3 at 100 TCID_50._ Following incubation at 36.5±1.5 °C for 70±10 min, the inoculum was removed and the cultures were refed with 2 ml/well maintenance medium. Cultures were maintained for a minimum period of 14 days, examined regularly and fed at least once per week. On day 14, supernatant from each well that did not exhibit CPE was inoculated onto fresh detector cells (blind passage). A further negative control was inoculated alongside the harvested supernatants. Following adsorption of inocula, the cultures were incubated for a further 14 days (a total of 28 days). During this period the cultures were examined regularly for CPE and fed at least once per week. The appearance of CPE was recorded and images were taken where appropriate. Data represent inoculations performed in 18 wells of detector cells seeded in 6-well plates, on three separate occasions (runs 1–3; Tables 1–3). Monolayers of cells in runs 1 and 2 were maintained for 28 days with a blind passage at day 14. Monolayers of cells in run 3 were maintained for 14 days.

### Statistical analysis

Statistical analysis for outliers were performed using GraphPad’s Online Grubbs test (significance level 0.05, two-tailed).
